# I WILL KEEP CONTRIBUTING TO MY FAMILY: FAMILY OBLIGATION AMONG OLDER ADULTS WITH MEXICAN IMMIGRANT BACKGROUNDS

**DOI:** 10.1093/geroni/igz038.1374

**Published:** 2019-11-08

**Authors:** Noriko Toyokawa

**Affiliations:** California State University San Marcos, San Marcos, California, United States

## Abstract

Providing care of older parents is a family obligation for children with Mexican cultural contexts (Knight et al., 2010). Nevertheless, little is known about how parents with Mexican cultural backgrounds believe about their family obligations. The current study conceptualized Mexican American older adults’ sense of family obligation. Data was collected from 307 Mexican Americans (Mage=54, SD=8, range 45-77 years old, females=56%) through an online survey. A 2-factor model: Expectation on children’s caregiving (3-item) and Efforts to reduce children’s burden (7-item) were identified as the best-fit model through EFA and CFA analyses (CFI=.96, SRMSA=.4). The component of efforts to reduce children’s burden predicted participants’ generativity assessed by the scale of McAdams and Aubin (1992). The findings suggest that Mexican American older adults expect their children to take care of them, as they feel obligated to reduce their children’s caregiving burden. The function of the cultural value in intergenerational relations is discussed.

